# Midline abdominal incision with left transverse extension for type IV thoracoabdominal aortic aneurysm repair

**DOI:** 10.1016/j.jvscit.2025.102062

**Published:** 2025-11-19

**Authors:** Yorihiko Matsumoto, Tsutomu Ito, Norimasa Haijima, Hirofumi Kasahara, Motohiko Osako, Hideyuki Shimizu

**Affiliations:** a Department of Cardiovascular Surgery, Keio University School of Medicine, Tokyo, Japan; b Department of Cardiovascular Surgery, Tokyo Dental College, Ichikawa General Hospital, Ichikawa, Japan; c Department of Cardiovascular Surgery, National Hospital Organization Saitama Hospital, Wako, Japan; d Department of Cardiovascular Surgery, Hiratsuka City Hospital, Hiratsuka, Japan; e Department of Cardiovascular Surgery, National Hospital Organization Tokyo Medical Center, Meguro, Japan

**Keywords:** Abdominal surgery, Crawford type IV, Suprarenal aortic aneurysm, Thoracoabdominal aortic aneurysm

## Abstract

We describe a technique for repairing Crawford type IV thoracoabdominal aortic aneurysms using a midline abdominal incision with left transverse extension, performed in the supine position without thoracotomy. Six patients (median age, 73 years) underwent this approach for true aneurysms, chronic dissection, or aortoduodenal fistula. Aortic reconstruction was performed with four-branched or bifurcated grafts under selective visceral perfusion. All patients were discharged home; one required reoperation for bleeding, and one developed an abdominal wall incisional hernia. This approach provided sufficient exposure for proximal and distal anastomoses and facilitated concurrent abdominal procedures while minimizing respiratory complications.

In repairing type IV thoracoabdominal aortic aneurysms (TAAAs) per Crawford’s classification,^1^ left lateral thoracotomy is often used.^2^^,^^3^ However, this approach has drawbacks, including a large thoracotomy, diaphragmatic incision, and right-side lateral position. We propose an alternative: a Midline Abdominal incision with Left Transverse extension (MALT approach) in a supine position, avoiding thoracotomy to preserve respiratory function and improve access to the iliac arteries and abdominal organs.

Patient consent was obtained from all patients who were included in this report.

## Technique

**Fig 1:**
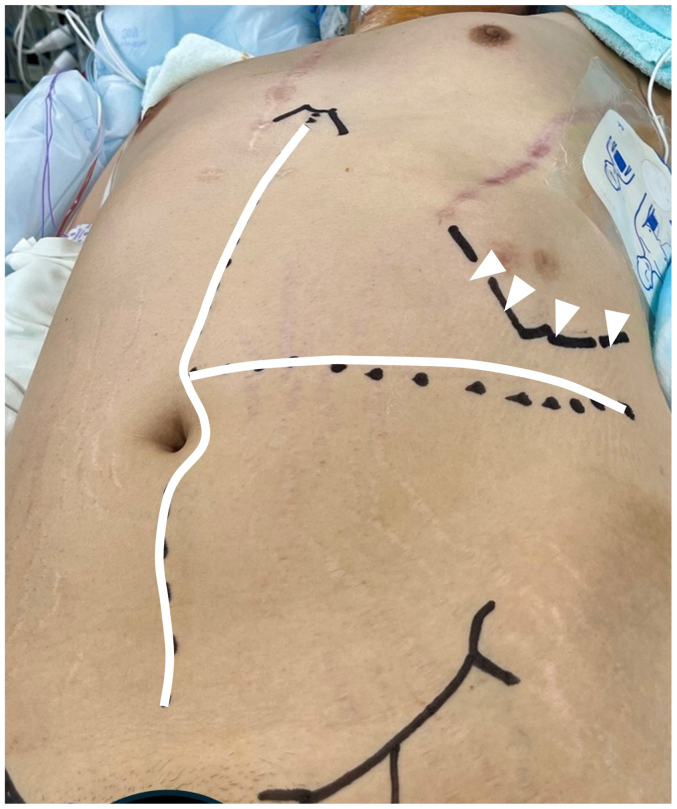
Preoperative patient positioning showing the T-shaped skin incision (*white line*) and the line of the left costal margin (*white arrowheads*).

**Fig 2:**
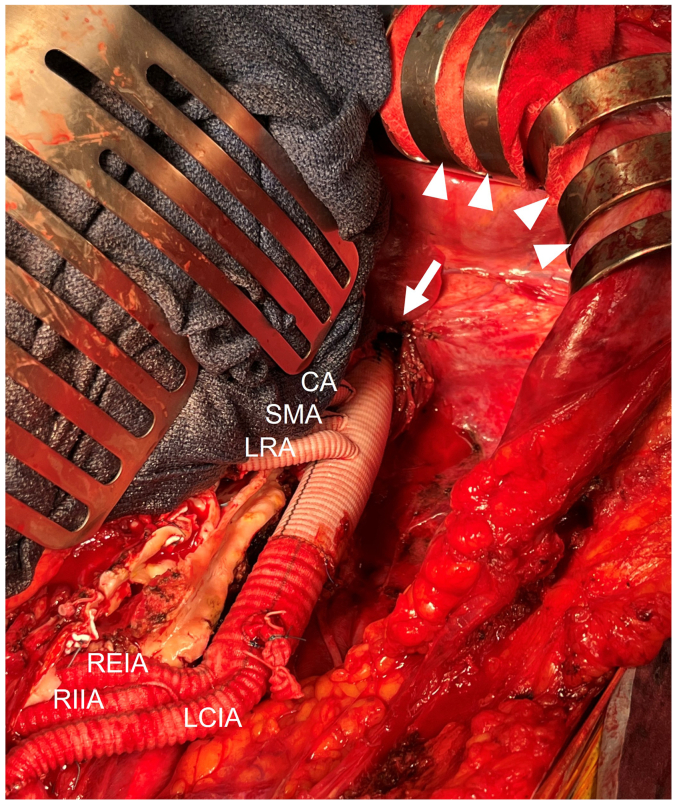
Intraoperative findings showing the incision of the left diaphragmatic crus and the proximal anastomosis (*white arrow*). The left costal margin is retracted cranially using Kent hooks (*white arrowheads*). *CA*, Celiac artery; *LCIA*, left common iliac artery; *REIA*, right external iliac artery; *RIIA*, right internal iliac artery; *RRA*, right renal artery; *SMA*, superior mesenteric artery.

In a supine position with their left arm raised anteriorly, a T-shaped laparotomy is made with a combination of a midline abdominal incision and a left-side transverse incision from the upper umbilicus to the lower margin of the left rib arch (Fig 1). The left costal arch is retracted cranially with a Kent hook (Fig 2). The retroperitoneal space is opened along the left paracolic gutter, and the abdominal aorta and the left common iliac artery are exposed. The proximal abdominal aorta is exposed by medial mobilization of the spleen and left kidney en bloc. The distal part of the descending aorta is exposed with the division of the left diaphragmatic crus (Fig 2). The right side of the iliac artery is exposed with a transperitoneal approach. During the aortic repair, the lower body and abdominal organs are perfused with partial cardiopulmonary bypass and selective visceral perfusion, established through groin access. A commercially available four-branched vascular graft is used for the reconstruction of the thoracoabdominal aorta, and a bifurcated graft is used for the abdominal aorta and iliac arteries. In the closure of the transverse incision, the muscle layers—including the external oblique, internal oblique, and transversus abdominis—are closed in one layer with single interrupted sutures using 0 polydioxanone sutures, while the anterior and posterior rectus sheaths are closed separately in the same way. The transverse suture line is joined with the midline suture of the linea alba in a 90-degree fashion.

## Results

This technique was used in six male patients with a median age of 73 years (range, 35-79 years) (Table). One was an emergency case due to an aortoduodenal fistula (ADF) (case 2). Two patients had a postdissection aortic aneurysm associated with Marfan syndrome (cases 1 and 3). The reasons for choosing this approach included previous thoracotomy in three cases, chronic lung disease in one case, and concomitant abdominal procedures in one case. The case with ADF required additional procedures, including duodenojejunostomy, cholecystectomy, and the creation of decompression gastrostomy and jejunostomy. The mean operative time was 645 ± 110 minutes. One case required re-exploration due to bleeding from the lumbar arteries, which was surgically controlled. One patient developed an abdominal wall incisional hernia postoperatively. All patients were successfully discharged home.

**Table:** Patients’ background and operative results

No.	Age, years	Type of aneurysm	Classification	Reconstructed visceral branches	Distal anastomosis site	Postoperative complications
1	35	cAD (MFS)	Crawford Type IV	CA, SMA, bilateral RAs	Right IAA + EIA, left CIA	Incisional hernia
2	72	True	Crawford Type IV	CA, SMA, bilateral RAs	Abdominal aorta	
3	48	cAD (MFS)	Crawford Type IV	CA, SMA, bilateral RAs	Right IAA + EIA, left CIA	Re-exploration for bleeding
4	74	True	Suprarenal AAA	bilateral RAs	Right IAA + EIA, left CIA	
5	79	True	Suprarenal AAA	SMA, bilateral RAs	Right CIA, left EIA	
6	74	True	Suprarenal AAA	CA, SMA, bilateral RAs	Right CIA, left CIA	

*CA,* Celiac artery; *cAD,* chronic aortic dissection; *CIA,* common iliac artery; *EIA,* external iliac artery; *IIA,* internal iliac artery; *MFS,* Marfan syndrome; *RA,* renal artery; *SMA,* superior mesenteric artery.

## Discussion

Among approaches for type IV TAAA repair,^4^^,^^5^ the left lateral thoracotomy is common for its excellent proximal aorta exposure but limits access to the contralateral iliac region and may be contraindicated in patients with prior thoracic surgery or pulmonary disease due to pulmonary risks. The transperitoneal approach via median laparotomy provides better distal access but less proximal exposure. Endovascular devices are a potential option but are under review in this country. We report an alternative method to access both the proximal and distal regions without thoracotomy, featuring several key advantages.

First, in the MALT approach, a left transverse extension of the median laparotomy will significantly enhance access to the upper abdominal aorta. This requires effective traction of the left costal margin with a Kent hook and careful mobilization of the spleen and left kidney en bloc (Fig 2). Moreover, the use of a roll towel under the left flank for elevation facilitates retroperitoneal exposure. The exposure of the distal descending aorta is achieved by incising the left crus of the diaphragm. Although prior thoracic surgery increases the risk of lung injury, careful dissection of the posterior mediastinum around the aorta can help preserve pleural integrity. This technique can avoid a wide split of the diaphragm, reducing the risk of diaphragm nerve injury. Preventing postoperative respiratory impairment is crucial, especially in patients with preoperative lung issues.^6^

Second, this approach enables more extensive and complex intra-abdominal arterial reconstruction and organ repair. In cases 1, 3, and 4, anastomoses on the distal right side were performed on the external and internal iliac arteries. Moreover, in the patient with an ADF in case 2, the patient concomitantly underwent complex abdominal organ repair by cooperating with the abdominal surgeons.

Limitations of this approach include cases that require proximal aortic control within the thoracic cavity. The complexity of the incision line may also complicate wound closure and postoperative management. The indication for this strategy should be discussed individually with each patient. Patients with a history of previous thoracic surgery or impaired respiratory function may be suitable for this method.

## Conclusions

The MALT strategy provides better access to the right iliac artery region and abdominal organs, while avoiding respiratory complications compared with the right lateral decubitus approach with left thoracotomy. This method offers an alternative technique for repairing type IV TAAAs in patients with preoperative respiratory impairment or expected complicated abdominal procedures.

## Funding

None.

## Disclosures

None.
